# The impact of recommending iron supplements to women with depleted iron stores in early pregnancy on use of supplements, and factors associated with changes in iron status from early pregnancy to postpartum in a multi-ethnic population-based cohort

**DOI:** 10.1186/s12884-023-05668-5

**Published:** 2023-05-13

**Authors:** Marthe-Lise Næss-Andresen, Anne Karen Jenum, Jens Petter Berg, Ragnhild Sørum Falk, Line Sletner

**Affiliations:** 1 Department of General Practice, Institute of Health and Society, University of Oslo, Blindern, P.O. Box 1130, 0318 Oslo, Norway; 2 General Practice Research Unit, Department of General Practice, Institute of Health and Society, University of Oslo, Oslo, Norway; 3 Department of Medical Biochemistry, Institute of Clinical Medicine, University of Oslo, Oslo, Norway; 4 Oslo Centre for Biostatistics and Epidemiology, Research Support Services, Oslo University Hospital, Oslo, Norway; 5 Department of Paediatric and Adolescents Medicine, Akershus University Hospital, Lørenskog, Norway; 6 Institute of Clinical Medicine, University of Oslo, Oslo, Norway

**Keywords:** Nutrition, Iron deficiency, Anaemia, Supplementation, Pregnancy, Ethnic minority, Maternal and Child health

## Abstract

**Background:**

We aimed to evaluate the impact of recommending supplementation to pregnant women with serum ferritin (SF) < 20 µg/L in early pregnancy on use of supplements, and to explore which factors were associated with changes in iron status by different iron indicators to 14 weeks postpartum.

**Methods:**

A multi-ethnic population-based cohort study of 573 pregnant women examined at mean gestational week (GW) 15 (enrolment), at mean GW 28 and at the postpartum visit (mean 14 weeks after delivery). Women with SF < 20 µg/L at enrolment were recommended 30-50 mg iron supplementation and supplement use was assessed at all visits. Change of SF, soluble transferrin receptor and total body iron from enrolment to postpartum were calculated by subtracting the concentrations at the postpartum visit from that at enrolment. Linear and logistic regression analyses were performed to assess associations between use of supplements in GW 28 and changes in iron status and postpartum iron deficiency/anaemia. Change of iron status was categorized into ‘steady low’, ‘improvement’, ‘deterioration’, and ‘steady high’ based on SF status at enrolment and postpartum. Multinomial logistic regression analyses were performed to identify factors associated with change of iron status.

**Results:**

At enrolment, 44% had SF < 20 µg/L. Among these women (78% non-Western European origin), use of supplements increased from 25% (enrolment) to 65% (GW 28). Use of supplements in GW 28 was associated with improved iron levels by all three indicators (*p* < 0.05) and with haemoglobin concentration (*p* < 0.001) from enrolment to postpartum, and with lower odds of postpartum iron deficiency by SF and TBI (*p* < 0.05). Factors positively associated with ‘steady low’ were: use of supplements, postpartum haemorrhage, an unhealthy dietary pattern and South Asian ethnicity (*p* ≤ 0.01 for all); with ‘deterioration’: postpartum haemorrhage, an unhealthy dietary pattern, primiparity and no use of supplements (*p* < 0.01 for all), and with ‘improvement’: use of supplements, multiparity and South Asian ethnicity (*p* < 0.03 for all).

**Conclusions:**

Both supplement use and iron status improved from enrolment to the postpartum visit among women recommended supplementation. Dietary pattern, use of supplements, ethnicity, parity and postpartum haemorrhage were identified as factors associated with change in iron status.

**Supplementary Information:**

The online version contains supplementary material available at 10.1186/s12884-023-05668-5.

## Background

Iron is crucial for numerous physiological and cellular processes, and iron deficiency (ID) has diverse health consequences [1]. Persistent ID will lead to depleted iron stores, usually defined by a low serum ferritin (SF) concentration [1], and to iron deficiency anaemia (IDA). Iron needs are tripled during pregnancy due to expansion of maternal red cell mass and growth of the fetus and placenta [2]. ID is associated with maternal fatigue, potentially poorer quality of life, increased risk of postpartum depression, and a higher risk to develop IDA [3]. Gestational anaemia is associated with maternal, perinatal and neonatal mortality, low birth weight and preterm birth [1, 4, 5]. In 2019, the global anaemia prevalence in pregnant women was 36.5%, a slight decrease from 2000 [6], and ID is estimated to account for half of the cases of anaemia [7]. In pregnancy, interpretations of SF values can be hampered by infections, inflammation and pregnancy related changes [3, 8], and soluble transferrin receptor (sTfR) and total body iron (TBI) have been suggested as more valid iron indicators [9, 10].

Iron supplementation programs are implemented in low-income countries to meet the WHO Global Nutrition Target from 2012 to reduce the prevalence of anaemia with 25% by 2025 [7]. There is international consensus to prevent gestational anaemia, and WHO recommends daily or intermittent iron supplementation, depending on the populations’ risk of maternal anaemia [11]. In high-income countries the guidelines differ, some recommend universal supplements from early pregnancy [12], while others recommend supplements only to women with ID or anaemia [5, 13–15]. The Norwegian recommendations for antenatal care have changed several times over the last decades. From 1995, women in early pregnancy were recommended to be screened by SF and haemoglobin (Hb) concentration, and iron supplementation was recommended if the SF concentration was below 60 µg/L [16]. In 2005, the guidelines were revised, and recommended to screen women by Hb concentration only, and further to recommend supplementation if anaemia was detected [17]. These were the guidelines during the data collection period of the present study. In 2018 guidelines were revised again, now recommending women to be screened by SF and Hb concentration at their first antenatal visit, and to recommend supplementation if SF concentration is below 70 µg/L [14]. The current guideline aims to prevent gestational and postpartum ID and IDA, and is supported by a Nordic systematic review concluding that 40 mg iron supplementations from gestational week (GW) 18-20 is an effective strategy to prevent ID in more than 90% and IDA in more than 95% of women at delivery and at 6-8 weeks postpartum [1]. How to ensure that women follow the recommendations and increase their use of iron supplements is less clear. Previous studies have shown that maternal age, socioeconomic status, parity, body mass index (BMI) and ethnic origin are factors affecting the use of iron supplements during pregnancy [18, 19]**.**

The STORK-G study included hard-to-reach-women, as all information, material and questionnaires were translated into eight languages, and about 20% of women with non-Western European origin were assisted by professional interpreters. We have previously described the prevalence of gestational and postpartum ID by different indicators and anaemia in our cohort [8, 20]. We found significantly higher prevalence of ID and anaemia at GW 15 in ethnic minority women compared to Western European women [8], while at the postpartum visit the ethnic disparities had almost disappeared [20]. We therefore wanted to assess factors associated with the change in iron status. Women with SF concentration < 20 µg/L at enrolment (GW 15), were provided written information describing their iron status and recommended 30-50 mg iron supplementation. The aims of this paper were therefore (1) to evaluate the impact of this simple recommendation of supplementation to pregnant women with depleted iron stores (SF < 20 µg/L) in early pregnancy on the self-reported use of supplements later in pregnancy in a population with diverse ethnic origin and socioeconomic status, and (2) to explore which factors were associated with changes in iron status by different iron indicators from enrolment to 14 weeks postpartum in women recommended supplementation or not.

## Subjects and methods

### Study population and data selection

Data from the population-based multi-ethnic STORK-G cohort were collected at public Child Health Clinics for primary antenatal care in three administrative districts in Oslo, Norway between 2008 and 2010. The study methods have been described in detail elsewhere [21]. In short, information, material and questionnaires were translated into Arabic, English, Sorani, Somali, Tamil, Turkish, Urdu and Vietnamese and quality checked by bilingual health professionals. Pregnant women were eligible if they (I) lived in the district, (II) planned to give birth at one of the two study hospitals, (III) were in < 20 GW, (IV) were not suffering from diseases necessitating intensive hospital follow-up during pregnancy, (V) could communicate in Norwegian or any of the eight specified languages and (VI) were able to provide written informed consent.

In total, 823 healthy women were enrolled at mean GW 15 ± standard deviation (SD) 3.4 in this cohort study; referred to as ‘at enrolment’ with planned follow-up visits in GW 28 (GW 28 ± 1.3) and approximately three months after delivery (13.9 ± 2.4 weeks after delivery), referred to as ‘postpartum visit’. Questionnaire data covering a wide range of health issues were collected through interviews by authorized study personnel, assisted by professional interpreters when needed at all three study visits [21]. In addition, clinical measurements were collected according to the study protocol. Participating women were found representative for the main ethnic groups of pregnant women attending the Child Health Clinics [21]. The study protocol and the consent-forms were approved by The Regional Committee for Medical and Health Research Ethics for South Eastern Norway and The Norwegian Data Inspectorate.

### Measurements of iron indicators (SF, sTfR and TBI) and Hb

Blood samples were drawn at all visits (2008-2011) [21] and sent the same day for analyses of SF and Hb at the Department of Multidisciplinary Laboratory Medicine and Medical Biochemistry at Akershus University Hospital, Lørenskog, Norway. SF concentration was measured using an electro-chemiluminescence immunoassay (ECLIA) method (Unicel DxI 800 from Beckman Coulter; inter-assay CV < 7%). Hb was measured using an SLS method (XE 5000 from Sysmex; inter-assay CV < 0.7%).

In 2016, sTfR was analysed by ELISA (Modular P800 from Roche; inter-assay CV < 5%), at the Department of Medical Biochemistry at Oslo University Hospital, Oslo, Norway, using biobanked serum samples. We calculated TBI according to Cook [22] from the ratio of sTfR concentration (by Flowers assay) to SF concentration: − [log_10_ (sTfR × 1000 ÷ SF) − 2.8229] ÷ 0.1207). To convert our Roche sTfR concentration to Flowers sTfR concentrations, we used the conversion equation Flowers sTfR = 1.5 × Roche sTfR + 0.35 mg/L [22].

### Recommendation of supplementation

For ethical reasons, and according to the protocol, women with depleted iron stores (SF < 20 µg/L) at enrolment were provided written information describing their SF concentration, recommended to start iron supplementation 30-50 mg/day, and to consult their General Practitioner (GP) for follow-up. At enrolment, in GW 28, and postpartum, all participants were asked about their intake of iron supplements during the past two weeks and to specify the name of the compounds used, and the number of tablets per day and per week. Of those reporting type of iron supplements, 57% used ferrous gluconate, 39% ferrous sulphate, and 4% ferrous fumarate. However, we were only able to calculate the exact iron intake in GW 28 in about 40% of cases. Iron supplementation was therefore dichotomized as ‘no intake’ and ‘intake of iron supplementation’, covering daily or intermittent iron supplement use.

### Outcome measures

Our outcome measures were postpartum ID (defined as SF concentration < 15 μg/L, sTfR concentration > 4.4 mg/L or TBI < 0 mg/kg) and anaemia (defined as Hb < 12.0 g/dL), and changes in SF, sTfR, TBI and Hb concentrations, calculated by subtracting the concentrations at the postpartum visit from that at enrolment for each indicator. Further, according to the SF concentration at enrolment the sample was first dichotomized, as either ‘recommended supplements’ (SF < 20 μg/L) or ‘not recommended supplements’ (SF ≥ 20 μg/L). Second, we further categorized women with SF > 20 μg/L into four groups (20-29, 30-49, 50-69, > 70 μg/L). Third, the sample was categorized based on the postpartum iron status (SF < 15 μg/L or ≥ 15 μg/L), resulting in four distinct groups called ‘steady low’, ‘improvement’, ‘deterioration’, and ‘steady high’, reflecting their change in iron status by SF from enrolment to postpartum.

### Sociodemographic variables

Maternal age was calculated from date of birth and date at enrolment in the present study. Parity was dichotomized into primiparous (first pregnancy lasting > 22 weeks) and parous (one or more previous births) women. Pre-pregnancy BMI (kg/m^2^) was calculated from self-reported weight before pregnancy and height measured at enrolment [21]. GW was primarily derived from the first day of the mother’s last menstrual period (LMP), but ultrasound-derived gestational age was used in 7% of pregnancies where there were reasons to believe the LMP-derived gestational age was uncertain [23]. Ethnicity was defined as the participant’s country of birth, or the participant’s mother’s country of birth if the participant’s mother was born outside Europe or North America [21], and grouped as Western Europeans (Norway, other Western European countries and North America), South Asians (primarily Pakistan and Sri Lanka), Middle Easterners (primarily Iraq, Morocco, and Turkey), East Asians (includes East- and South-East Asian countries, primarily Vietnam and The Philippines), Sub-Saharan Africans (primarily Somalia) and Eastern Europeans (primarily Poland, Kosovo, and Russia). Maternal present socioeconomic position (SEP) was a score derived from a principal component analysis (PCA) of 11 different demographic variables [24]. The variables contributing most to the score were individual level data about education, occupational class and employment status, and household variables as own or renting tenure and rooms per person in the household. The score was standard normally distributed, with a higher score reflecting higher SEP.

### Variables potentially associated with iron metabolism

From questions about the women’s medical history, we categorized three groups; (I) no medical conditions associated with anaemia or ID, (II) self-reported chronic illness or medication associated with ID or normochromic, and (III) self-reported chronic illness or medication associated with ID or hypochromic anaemia [8]. Data from a food frequency questionnaire, developed to capture dietary patterns in a multi-ethnic sample, were collected in GW 28. Four clusters were extracted using the Ward’s method [25]. Clusters were referred to as ‘a healthier dietary pattern’ vs. three ‘less healthy dietary patterns’ [25], here dichotomized into ‘healthy’ and ‘unhealthy’. The ‘healthy dietary pattern’ represented more frequent intake of fruit, vegetables, wholegrain bread with pate and meat spread, and meat, i.e. food items representing relatively high iron content (heme and non-heme) and foods rich in vitamin C which again improves the bioavailability of iron compared to the other dietary pattern. After conducting sensitivity analyses excluding women with possible inflammation (elevated C-reactive protein (CRP)) in our previous published papers [8, 20], we found only minor changes in the mean and median values and in the prevalence of ID and anaemia across ethnic groups and concluded that inflammation did not explain the differences observed in our population. We therefore chose not to adjust SF, sTfR or TBI values for CRP to correct for inflammation, as suggested by some others [26–28].

### Birth-related variables potentially associated with postpartum iron status

We have detailed data on birth complications extracted from hospital birth records. Delivery mode was categorized as normal vaginal delivery, instrumental vaginal delivery (i.e. forceps or vacuum assisted vaginal delivery), elective caesarean section and emergency caesarean section. Blood loss after delivery was dichotomized into < 500 mL and ≥ 500 mL, where the latter was defined as postpartum haemorrhage. We constructed a composite index for birth complications reflecting the presence of at least one of the following complications; episiotomy, third- or fourth-degree perineal tear, obstructed labour and manual removal of placenta, due to small numbers in each of the categories.

### Sample size

Of the 823 women enrolled in mean GW 15, 644 (78%) women gave birth to a live and singleton baby and attended the postpartum visit. For this study, we included participants with values for SF from both visits, resulting in a total sample of 573 women (Flow chart, Additional file 1, Figure S1). There were no significant differences between the study sample and the 250 excluded women for age, parity, pre-pregnant BMI, and SEP (data not shown). However, the study sample consisted of a slightly larger proportion of ethnic minority women compared to the excluded women, as they were prioritised for fasting blood samples at the postpartum visit due to resource limitations [20].

### Statistical analyses

Descriptive statistics are presented as frequencies with proportions for categorical variables and mean with SD or medians with interquartile range for continuous variables. The sTfR and TBI values were approximately normally distributed, and SF skewed to the left. Difference in SF from enrolment to postpartum within the five SF categories at enrolment was assessed by Wilcoxon signed rank test.

We performed multinomial logistic regression analyses to investigate factors associated with belonging to one of the following three groups ‘steady low’, ‘improvement’ and ‘deterioration’, reflecting the women’s iron status at enrolment and postpartum, using ‘steady high’ as the reference. Both unadjusted and adjusted odds ratios (OR) are presented. GW, age, parity, SEP, diet, ethnicity, use of iron supplementation in GW 28 and postpartum, postpartum haemorrhage and birth complications were included in the fully adjusted model.

Furthermore, we stratified the cohort in two subsamples based on whether the women were recommended use of iron supplements at enrolment or not. In each stratum, we performed two sets of regression analyses. Linear regression analyses were performed to assess the association between self-reported use of iron supplementation in GW 28 and changes in SF, sTfR, TBI, and Hb concentrations from enrolment to the postpartum visit. Logistic regression analyses were performed when postpartum ID (defined by SF, sTfR and TBI) and anaemia were the outcome. Adjustments were made for GW, age, parity, SEP, diet, ethnicity, and use of iron supplements in pregnancy. In addition, SF concentration at enrolment was included to reduce dilution of regression to the mean. In a final model we also adjusted for use of supplements postpartum, postpartum haemorrhage, and birth complications although these were not true confounders, but potentially strongly associated with the outcome. The estimates changed marginally by including the last covariates. Results are presented as β-coefficients and ORs with accompanied 95% confidence intervals (CI). SPSS version 28 and Stata version 16 were used for statistical analysis.

## Results

At enrolment (mean GW 15.1 (SD ± 3.4), maternal age was 29.7 ± 4.8 years, pre-pregnant BMI was 24.6 ± 4.8 kg/m^2^, 45% were primiparous and 62% had ethnic origin from countries outside Western Europe (Table 1). Iron supplementation was recommended to 252 (44%) women with depleted iron status (SF < 20 µg/L) at enrolment in early pregnancy, of whom 204 (78%) were of non-Western European origin.

**Table 1:** Characteristics of participants by serum ferritin concentration at enrolment and postpartum ^1^

	n	Total	SF < 20 μg/L in early pregnancy (depleted iron stores and recommended supplements)			SF ≥ 20 μg/L in early pregnancy (not exposed to recommendations)		
			Total	SF < 15 μg/l postpartum	SF ≥ 15 μg/l postpartum	Total	SF < 15 μg/L postpartum	SF ≥ 15 μg/L postpartum
				Steady low	Improvement		Deterioration	Steady high
		*n* = 573	*n* = 252 (44)	*n* = 111	*n* = 141	*n* = 321 (56)	*n* = 114	*n* = 207
Mean baseline SF (95% CI)		33 (30, 35)	11 (11, 12)	11 (10, 12)	12 (11, 12)	50 (46, 54)	45 (40, 50)	53 (47, 58)
Median baseline SF (IQR)		23 (12, 41)	11 (7,15)	11 (7, 15)	12 (8, 15)	38 (27, 60)	34 (26, 55)	40 (27, 62)
Gestational week at enrolment	573	15.1 ± 3.4	16.1 ± 3.9	15.7 ± 3.8	16.5 ± 4.0	14.2 ± 2.6	14.2 ± 2.8	14.3 ± 2.6
Postpartum week	565	13.9 ± 2.4	14.0 ± 2.5	14.0 ± 2.7	14.0 ± 2.4	13.9 ± 2.4	13.6 ± 2.4	14.0 ± 2.4
Parous women (≥ 1 previous births)	573	313 (55)	163 (65)	69 (63)	94 (67)	150 (47)	36 (32)	114 (55)
Age at inclusion,* years*	573	29.7 ± 4.8	29.4 ± 4.9	29.1 ± 5.3	29.6 ± 4.6	30.0 ± 4.8	29.3 ± 4.9	30.3 ± 4.6
Pre-pregnant Body Mass Index, kg/m2	563	24.6 ± 4.8	23.9 ± 4.5	23.9 ± 4.3	23.8 ± 4.7	25.3 ± 4.9	25.4 ± 5.6	25.2 ± 4.5
Western European ethnicity	217	217 (38)	48 (22)	17 (15)	31 (22)	170 (78)	61 (54)	109 (53)
South Asian ethnicity	157	157 (27)	102 (65)	46 (42)	56 (40)	55 (35)	23 (20)	32 (15)
Middle Eastern ethnicity	94	94 (16)	51 (54)	11 (10)	12 (9)	43 (46)	7 (6)	7 (3)
Sub-Saharan African ethnicity	37	37 (7)	23 (62)	5 (5)	5 (4)	14 (38)	7 (6)	16 (8)
East Asian Ethnicity	33	33 (6)	10 (30)	5 (5)	5 (4)	23 (70)	7 (6)	16 (8)
Eastern European ethnicity	34	34 (6)	18 (53)	7 (6)	11 (8)	16 (47)	4 (4)	12 (33)
Socioeconomic position ^2^	569	0.02 (1.0)	-0.2 (1.0)	-0.4 (1.0)	-0.1 (1.0)	0.2 (1.0)	0.2 (0.9)	0.3 (1.0)
Unhealthy dietary pattern ^3^	555	391 (70)	202 (82)	95 (86)	107 (76)	189 (61)	80 (70)	109 (53)
Chronic illness / medication associated with normochromic anaemia ^4^	564	14 (2.5)	4 (1.6)	3 (3)	1 (1)	10 (3.2)	4 (4)	6 (3)
Chronic illness / medication associated with hypochromic anaemia ^5^	564	55 (10)	24 (10)	10 (90)	14 (10)	31 (10)	8 (7)	23 (11)
Self-reported use of iron supplement in early pregnancy	564	101 (18)	61 (25)	24 (22)	37 (26)	40 (13)	16 (14)	24 (12)
Self-reported use of iron supplement in gestational week 28	538	232 (43)	156 (65)	60 (55)	96 (68)	76 (26)	17 (15)	59 (29)
Self-reported use of iron supplement 14 weeks postpartum	554	124 (22)	69 (28)	25 (23)	44 (31)	55 (18)	20 (18)	35 (17)
Normal vaginal delivery	414	414 (72)	188 (75)	78 (71)	110 (78)	226 (70)	76 (67)	150 (73)
Instrumental vaginal delivery	58	58 (10)	29 (12)	16 (15)	13 (9)	29 (9)	11 (10)	18 (9)
Elective caesarean section	68	68 (12)	25 (10	12 (11)	13 (9)	43 (13)	18 (16)	25 (12)
Emergency caesarean section	29	29 (5)	8 (3)	5 (4)	4 (3)	21 (7)	7 (6)	14 (7)
Birth complications ^7^	573	97 (17)	35 (14)	15 (14)	20 (14)	62 (19)	30 (26)	32 (15)
Postpartum haemorrhage (≥ 500 mL)	573	33 (5.7)	12 (4.8)	8 (7)	4 (3)	21 (6.5)	13 (11)	8 (4)

*Enrolment* mean gestational week 15; *GW* Gestational week, *Hb* Haemoglobin, *Postpartum* mean 14 weeks after delivery, *SF* Serum ferritin

^1^ The STORK-G multi ethnic pregnancy cohort from Oslo, Norway, 2008-2010. Values are n (%) or mean ± standard deviation. Group designation: Steady low, SF < 20 μg/L at enrolment and < 15 μg/l postpartum; Improvement, SF < 20 μg/L at enrolment and SF ≥ 15 μg/l postpartum; Deterioration, SF ≥ 20 μg/L at enrolment and SF < 15 μg/l postpartum, and Steady high, SF ≥ 20 μg/L at enrolment and SF ≥ 15 μg/l postpartum

^2^ Maternal present socioeconomic position (SEP) was a score derived from a principal component analysis (PCA) of 11 different demographic variables. The variables contributing most to the score, were individual level data about education, occupational class and employment status, and household variables as own or renting tenure and rooms per person in the household. The score was normally distributed, had mean = 0 and SD = 1 (mean and range), with a higher score reflecting higher SEP

^3^ Self-reported dietary pattern in gestational week 28 extracted from a food frequency questionnaire

^4^ Self-reported chronic illness or medication associated with normochromic anaemia (i.e. kidney or rheumatic disease, use of carbamazepine or infliximab)

^5^ Self-reported chronic illness or medication associated with ID and hypochromic anaemia (i.e. gastrointestinal disease or Copper intrauterine device use before conception)

^6^ Forceps or vacuum assisted vaginal delivery

^7^ A composite variable created by combining following 4 birth complication; episiotomy, third—and fourth degree perineal tear, obstructed labour, and manual removal of placenta

From enrolment in early pregnancy to GW 28, the reported use of iron supplementation increased from 25 to 65% in women recommended supplements, and from 13 to 26% in the group not exposed to our recommendations. Further, the proportion of women reporting use of iron supplements at GW 28 was higher (68%) in the group with ‘improvement’ in iron status from enrolment in early pregnancy to postpartum (mean postpartum week 13.9 (SD ± 2.5), compared to the group with ‘steady low’ iron status (55%). It was also higher in women with ‘steady high’ (29%) compared to the group with ‘deterioration’ (15%) in iron status (Table 1**).**

### The effect of supplemental use

**Fig. 1:**
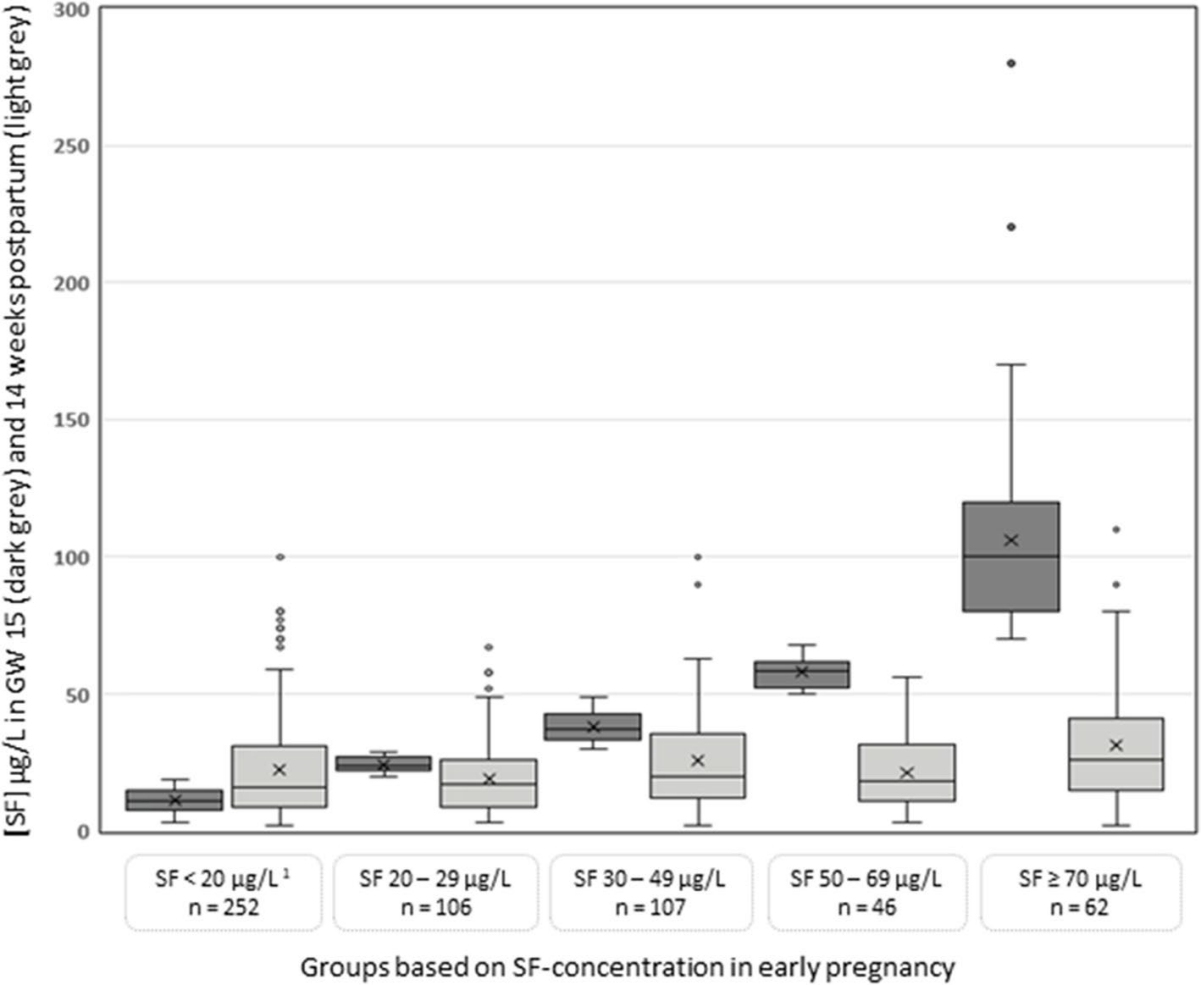
Change in serum ferritin concentration from enrolment in early pregnancy to postpartum in participants with different value at enrolment ^1^. ^1^ Data from the STORK-G multi-ethnic pregnancy cohort from Oslo, Norway, 2008-2010. The group with SF < 20 μg/L (depleted iron stores) at enrolment, were recommended supplementation. *Enrolment* mean gestational week 15.1 ± standard deviation 3.4. *Postpartum visit* mean postpartum week 13.9 ± 2.4. SF, serum ferritin

The change in SF concentration from enrolment in early pregnancy to the postpartum visit, stratified into five groups by SF concentration at enrolment, is illustrated in Fig. 1. A moderate increase in median SF concentration was observed in women recommended iron supplementation (*p* < 0.001), while a reduction was seen in all other groups (*p* < 0.001 in all). As a consequence, the prevalence of postpartum ID by SF (defined as SF < 15 µg/L) was approximately 40% in all groups, except in women with SF concentration ≥ 70 µg/L at enrolment (24%) (Additional file 2, Table S1). The prevalence of postpartum ID in the group recommended iron supplementation (SF < 20 μg/L at enrolment) was 44% by SF, 25% by sTfR and 28% by TBI, while 31% had postpartum anaemia** (**Additional file 2, Table S1). The change in SF from enrolment in early pregnancy to the postpartum visit shown in Fig. 1 were similar across the largest ethnic groups (data not shown)*.*

Results from the multinomial logistic analysis showed that compared to ‘steady high’ iron status by SF, use of supplements in GW 28 was positively associated with having ‘steady low’ (OR 2.7, 95% CI 1.5-4.8, *p* < 0.001), but even more strongly with ‘improvement’ (OR 5.2, 95% CI 3.0-9.0,* p* < 0.001) in iron status by SF, while negatively associated with having ‘deterioration’ in iron status by SF (OR 0.3, 95% CI 0.2-0.6, *p* < 0.001) (Table 2).

**Table 2:** Factors associated with change in serum ferritin from enrolment in early pregnancy to postpartum visit ^1^

	SF < 20 μg/L at enrolment (depleted iron stores and recommended supplements)												SF ≥ 20 μg/L at enrolment (not exposed to recommendations)					
	Steady low						Improvement						Deterioration					
	*n* = 111						*n* = 141						*n* = 114					
Candidate factors	Postpartum iron deficiency (SF < 15 μg/L)						Postpartum *no* iron deficiency SF ≥ 15 μg/L						Postpartum iron deficiency (SF < 15 μg/L)					
	Unadjusted model			Final model			Unadjusted model			Final model			Unadjusted model			Final model		
	OR	95% CI	*p*	Adj OR	95% CI	*p*	OR	95% CI	*p*	Adj OR	95% CI	*p*	OR	95% CI	*p*	Adj OR	95% CI	*p*
Self-reported use of iron supplement in GW 28	3.0	1.8–4.9	< 0.001	2.7	1.5–4.8	< 0.001	5.2	3.2–8.3	< 0.001	5.2	3.0–9.0	< 0.001	0.4	0.2–0.8	0.004	0.3	0.2–0.6	0.001
Unhealthy dietary pattern ^2^	7.0	3.5–13.8	< 0.001	4.0	1.7–9.5	0.001	2.7	1.7–4.4	< 0.001	1.5	0.8–3.0	0.212	2.0	1.2–3.2	0.008	2.8	1.5–5.2	0.002
Primiparous women	0.7	0.4–1.1	0.160	0.8	0.4–1.5	0.447	0.6	0.4–1.0	0.031	0.5	0.3–0.9	0.033	2.7	1.6–4.3	< 0.001	2.7	1.5–5.1	0.002
Postpartum haemorrhage (≥ 500 mL)	2.0	0.7–5.3	0.197	5.4	1.5–19.2	0.010	0.7	0.2–2.5	0.607	1.7	0.4–6.9	0.486	3.2	1.3–8.0	0.012	4.7	1.6–14.0	0.006
Western European ethnicity (reference)																		
South Asian ethnicity	9.8	4.9–19.6	< 0.001	3.7	1.6–8.7	0.003	6.2	3.4–11.1	< 0.001	2.7	1.2–5.9	0.014	1.3	0.7–2.3	0.429	0.7	0.1–0.8	0.295
Middle Eastern ethnicity	5.5	2.6–11.6	< 0.001	2.4	0.9–6.1	0.072	2.9	1.5–5.7	0.001	1.8	0.7–4.3	0.796	0.7	0.3–1.4	0.327	0.3	0.1–0.8	0.021
Sub-Saharan African ethnicity	10.7	3.6–31.6	< 0.001	2.6	0.7–10.4	0.173	6.0	2.2–16.6	< 0.001	2.7	0.7–4.3	0.146	1.8	0.6–5.3	0.298	1.1	0.3–3.9	0.927
East Asian Ethnicity	2.1	0.7–6.6	0.191	1.0	0.3–3.7	0.965	1.1	0.4–3.2	0.864	0.6	0.2-.2.3	0.477	0.8	0.3–2.0	0.608	0.6	0.2–1.8	0.361
Eastern European ethnicity	4.0	1.3–11.6	0.011	1.8	0.5–6.5	0.326	3.2	1.3–8.0	0.012	2.2	0.8–6.6	0.145	0.6	0.2–1.9	0.387	0.3	0.1–1.2	0.091

*Adj* Adjusted, *enrolment* mean gestational week 15, *GW* Gestational week, *OR* Odds ratio, *postpartum* mean 14 weeks after delivery, *postpartum ID by SF* serum ferritin < 15 µg/L, *SF* Serum ferritin, *95% CI* 95% confidence interval

^1^ Multinomial logistic regression analyses to investigate factors associated with belonging to one of the following three groups ‘steady low’, ‘improvement’ and ‘deterioration’, reflecting the women’s iron status by serum ferritin at enrolment and postpartum, using ‘steady high’ (n = 207) as the reference in the STORK-G study, a multi-ethnic pregnancy cohort from Oslo, Norway, 2008-2010

^2^ Both unadjusted and adjusted odds ratios are presented. The fully adjusted model included gestational week, age, parity, socioeconomic position, diet, ethnicity, use of iron supplementation in gestational week 28 and postpartum, postpartum haemorrhage and birth complications

^3^ Self-reported dietary pattern in gestational week 28 extracted from a food frequency questionnaire

The stratified linear and logistic regression analyses showed that use of iron supplements in GW 28 was associated with improvement in iron status from enrolment in early pregnancy to 14 weeks postpartum by SF, sTfR, TBI, and in Hb concentration (*p* < 0.05—0.001) **(**Table 3**).** Use of supplements were associated with lower odds of postpartum ID by SF and TBI (*p* < 0.05—0.001) both in women exposed to recommendations and not. However, supplement use in iron depleted women (SF < 20 µg/L at enrolment) was not significantly associated with lower odds of postpartum ID by sTfR, and supplement use was in neither groups significantly associated with lower odds of postpartum anaemia (Table 3). Both SF change and TBI change were consistently associated with supplementation use.

**Table 3:** Use of supplementation on changes in iron status and odds of postpartum iron deficiency and anaemia ^1^

Linear regression: change in SF, sTfR, TBI, and Hb concentration							Logistic regression: postpartum iron deficiency or anaemia						
	Unadjusted model			Final linear model ^a^				Unadjusted model			Final logistic model ^a^		
	β	95% CI	*p*-value	Adj β	95% CI	*p*-value		OR	95% CI	*p*-value	Adj OR	95% CI	*p*-value
Recommended supplements (SF < 20 μg/L at enrolment in early pregnancy)							Recommended supplements (SF < 20 μg/L at enrolment in early pregnancy)						
Self-reported use of supplementation in GW 28							Self-reported use of supplementation in GW 28						
Change in SF	5.7	1.0, 10	0.018	5.1	0.2, 10.0	0.040	Postpartum ID by SF	0.6	0.3, 1.0	0.047	0.5	0.3, 1.0	0.044
Change in sTfR	-0.7	-1.2, -0.3	0.003	-0.7	-1.2, -0.2	0.009	Postpartum ID by sTfR	0.9	0.5, 1.6	0.665	0.6	0.3, 1.3	0.221
Change in TBI	2.1	1.1, 3.2	< 0.001	1.8	0.7, 2.9	0.001	Postpartum ID by TBI	0.4	0.2, 0.8	0.005	0.3	0.1, 0.6	0.001
Change in Hb	0.5	0.3, 0.8	< 0.001	0.6	0.4, 0.9	< 0.001	Postpartum anaemia	0.8	0,5, 1.5	0.560	0.7	0.4, 1.4	0.348
SF ≥ 20 μg/L at enrolment in early pregnancy (not exposed to recommendations)							SF ≥ 20 μg/L at enrolment in early pregnancy (not exposed to recommendations)						
Self-reported use of iron supplementation in GW 28							Self-reported use of supplementation in GW 28						
Change in SF	18.6	10, 21	< 0.001	5.1	0.5, 9.6	0.029	Postpartum ID by SF	0.4	0.2, 0.8	0.004	0.4	0.2, 0.7	0.004
Change in sTfR	-0.5	-0.9, -0.1	0.011	-0.6	-1.0, -0.2	0.007	Postpartum ID by sTfR	0.4	0.1, 0.9	0.039	0.3	0.91, 0.9	0.027
Change in TBI	2.2	1.3, 3.2	< 0.001	1.8	0.9, 2.7	< 0.001	Postpartum ID by TBI	0.3	0.1, 0.7	0.006	0.3	0.1, 0.6	0.001
Change in Hb	0.3	0.1, 0.6	0.010	0.4	0.2, 0.7	0.001	Postpartum anaemia	1.1	0.6, 2.0	0.854	0.9	0.5, 19	0.854

*Adj* Adjusted, *β* beta, *95% CI* 95% confidence interval, *enrolment*: mean gestational week 15, *GW* Gestational week, *Hb* Haemoglobin, *ID* Iron deficiency, *OR* Odds ratio, *postpartum* mean 14 weeks after delivery, *postpartum ID by SF* serum ferritin < 15 µg/L, *postpartum anaemia* Haemoglobin < 12.0 g/dL, *postpartum ID by sTfR* soluble transferrin receptor  > 4.4 mg/L, *postpartum ID by TBI* total body iron < 0 mg/kg, *SF* Serum ferritin, *sTfR* Soluble transferrin receptor, *TBI* Total body iron

^a^ Linear regression analyses to assess the association between use of supplementation in gestational week 28 and changes in SF, sTfR, TBI and Hb from enrolment to 14 weeks postpartum in iron depleted women (SF < 20 μg/L) recommended supplements at enrolment (top panel) or not (bottom panel) in the STORK-G, a multi-ethnic pregnancy cohort from Oslo, Norway, 2008-2010

^b^ Logistic regression analyses to assess the association between use of supplementation in gestational week 28 on iron deficiency by SF, sTfR, TBI and anaemia in iron depleted women (SF < 20 μg/L) recommended supplements at enrolment (top panel) or not (bottom panel) in the STORK-G, a multi-ethnic pregnancy cohort from Oslo, Norway, 2008-2010

^c^ Both unadjusted and adjusted beta and odds ratios are presented. The fully adjusted model included SF concentration at enrolment, gestational week, age, parity, socioeconomic position, diet, ethnicity, and use of supplement in gestational week 28

### Factors associated with change in iron status

In addition to no use of supplements, an unhealthy dietary pattern (ORs 4.0 and 2.8, *p* < 0.01) and postpartum haemorrhage (ORs 5.4 and 4.7, *p* ≤ 0.01) were associated with higher odds of having ‘steady low’ and ‘deterioration’ of iron status by SF (Table 2). Primiparity was associated with lower odds of ‘improvement’ (OR 0.5, *p* = 0.03) and higher odds of ‘deterioration’ (OR 2.7,* p* < 0.01) in iron status by SF. Further, South Asian ethnic origin was associated with having high odds of ‘improvement’ in iron status by SF, but higher odds of having ‘steady low’’ in iron status by SF (ORs 2.7 and 3.7, *p<0.02*) (Table 2).

## Discussion

### Main findings

In this cohort, iron supplementation was recommended to 44% of the pregnant women due to depleted iron status (SF concentration < 20 µg/L) at enrolment in mean GW 15, of whom the majority (78%) were of non-Western European origin. In the group recommended supplementation, the use of iron supplements increased from 25% at enrolment to 65% at the postpartum visit. Further, in women recommended supplements, median SF concentration increased from 11 to 16 µg/L, while the median SF concentration was substantially reduced in women not exposed to our recommendations. Use of iron supplements in GW 28 was associated with lower odds of ‘deterioration’ and higher odds of ‘improvement’ in iron status by SF, and with lower odds of postpartum ID by SF and TBI, both in women recommended iron supplement and those not exposed to our recommendations. Use of supplements in GW 28 did however not reduce the odds of postpartum anaemia or postpartum ID by sTfR in women with depleted iron stores (SF concentration < 20 µg/L) at enrolment. Primiparity, unhealthy dietary pattern and postpartum haemorrhage were independently associated with ‘steady low’ and ‘deterioration’ in iron status by SF. South Asian ethnic origin was associated with higher odds of both ‘steady low’ and ‘improvement’ of iron status by SF.

This study confirms the widespread prevalence of depleted iron stores in pregnancy and the early postpartum period [29]. Even though the prevalence of gestational anaemia in the total sample was low (5.9%) in a global context [8], as many as 89% had SF < 70 µg/L at enrolment, and would be recommended supplementation according to current Norwegian antenatal guidelines, implemented after this study was performed. We found that women not exposed to our recommendations showed a more adverse iron status development, and that women having SF between 20-70 µg/L at enrolment had a high prevalence of postpartum ID supports the current guidelines [14].

In our multi-ethnic cohort, we observed that 65% of the women recommended supplementation seemed to follow our recommendations and reported use in GW 28, and the median SF and the mean TBI concentration in this group increased from enrolment to 14 weeks postpartum. The compliance in randomized, controlled trials (RCT’s) has been reported to be around 85-90% [29–31], however strict follow-ups to encourage and ensure compliance might not reflect real-life settings. Previous studies have found a higher proportion of ID in populations with low socioeconomic status [32] and that pregnant women with ethnic minority background are less likely to follow recommendations regarding supplemental use [18]. Despite our recommendations, the proportion of postpartum ID in women recommended supplementation in our study was still high (33% by SF < 12 µg/L and 44% by SF < 15 µg/L). This is higher than observed in two RCT’s with low-dose iron supplementations, where the prevalence of ID (SF < 15 µg/L) 2-6 months after delivery was 7-16% in the intervention and 29% in the placebo group [29, 30]. Moreover, in an US cross-sectional study, 13% had ID (SF < 12 µg/L) 0-6 months after delivery [33]. There may be several reasons for such discrepancies. Our study was population-based, and it is well-known that participants in RCTs often are highly selected and motivated for the intervention. Furthermore, our cohort consists of many women from ethnic minority- and hard-to-reach groups, and we observed significant ethnic differences in iron status, including Hb levels at enrolment [8]. However, at the postpartum visit, only women of South Asian origin had significantly higher sTfR and lower Hb concentration [20] compared to Western Europeans, suggesting that the simple intervention recommending supplementation to iron depleted women, of whom 78% were ethnic minority women, had an effect on decreasing ethnic differences. The effects of iron supplements on change in SF from enrolment to the postpartum visit showed in Fig. 1 were similar across the largest ethnic groups (data not shown), indicating that these are universal effects.

In line with others, we found that use of supplements reduced the risk of postpartum ID and that women reporting use of iron supplements were more likely to improve their iron status by SF and mean Hb concentration in the postpartum period [1, 5]. We also found that supplementation was associated with improvements of iron status in mean sTfR and TBI. Nevertheless, while use of supplements in GW 28 was strongly associated with postpartum ID by TBI, it did not reduce the odds of postpartum anaemia, nor of postpartum ID by sTfR, in women with depleted iron stores (SF concentration < 20 µg/L) at enrolment. This could possibly suggest that TBI is a better indicator of the effect of supplementation use on ID. However, the test is more expensive, which restricts its general availability, and more data on its use in pregnancy are needed. Furthermore, SF in pregnancy is affected by haemodilution. We can hence not rule out the possibility that some of the increase in SF from enrolment to postpartum in women with depleted iron stores at baseline could be related to changes in blood volume. However, the observed decrease in SF observed in women that were not exposed to our recommendations, does not support this. Further, sTfR and TBI, which are less affected by pregnancy-related factors, show similar pattern, which suggests that changes in iron status measured by SF cannot be explained by pregnancy related changes such as haemodilution alone. Although associated with higher Hb concentration postpartum, use of supplements was in our cohort not associated with reduced risk of anaemia. A study from Burkina Faso, Africa, in a population with a high rate of anaemia while unknown iron status, found that iron supplementation was only associated with increasing Hb from early to late pregnancy if the woman was anaemic [34]. We did not have statistical power to evaluate the effect of iron supplements stratified by anaemia status, as few women were anaemic at enrolment (5.9%). Further, we were primarily interested in change in iron status by different iron indicators, and factors associated with such change.

In high-income countries, haemorrhage is recognized as the most important factor associated with postpartum ID and anaemia [1]. Our study supports that it is important to offer women with haemorrhage a postpartum follow-up to assess iron status. In line with others we found that primiparity appears to be an important factor associated with an adverse iron status development [35], also after adjusting for birth complications, haemorrhage, ethnicity and supplement use. This might suggest that there may be other, unidentified factors related to primiparous pregnancies that could affect the need for iron. We have previously reported significant ethnic differences in crude ID and anaemia prevalence. The prevalence of anaemia ranged from 7-14% in early pregnancy in women with ethnic origin outside Europe [8] and 26-40% postpartum [20]. Wheras in Europeans the prevalence was lower, both in early pregnancy (0-1.8%) [8] and postpartum (14-18%) [20]. Recent studies from the UK found a high proportion of women from ethnic minority groups to be anaemic during pregnancy, and also that severe anaemia was associated with adverse foetal and infant outcomes (stillbirth, perinatal death, small for gestational age infants, low birth weight infants and maternal postpartum haemorrhage [36]), highlighting the importance of preventing anaemia and identifying vulnerable groups, e.g. minority women, which could need extra attention. On the other hand, no existing evidence indicates that preventive iron supplementation has an effect on birth weight or other adverse infant outcomes [37]. In our study, 65% of women with South Asian ethnic origin had depleted iron stores at enrolment and were recommended supplementation. Many of these women benefitted from the simple intervention, having higher odds for being in the ‘improvement’ group, but some did not and developed postpartum ID (‘steady low’ group). This could suggest that language skills and cultural factors not accounted for in our study may also play a role in the compliance to our recommendations. Another possible explanation could be that this ethnic group have a diet richer of phytates, which inhibits iron uptake [38]. Therefore, clinicians should strive to increase the compliance in order to ensure improvement of iron status, prevent anaemia and to prevent associated adverse maternal and neonates outcome [5, 32, 36].

### Strength and limitations

The major strength of our multi-ethnic population-based study is that we followed a large number of healthy women from enrolment in early pregnancy to 14 weeks postpartum with measurements of iron status by three different indicators, analysed at the same laboratories. We collected a broad high-quality data set, which enabled us to explore relations between iron status and a wide range of explanatory factors, and to adjust for a range of possible confounders. The questionnaires were translated to eight languages and data collection methods were adapted to facilitate enrolment of ethnic minorities and even illiterate women, who often are excluded in research [21]. Professional interpreters were used to ensure the quality of the interview-administrated questionnaire data. The women were found representative for the main ethnic groups of Oslo. Lastly, this study represents close-to-practice research, addressing the gap between what works in research (RCT) and what works in practice, especially in hard-to-reach-groups.

However, there are also weaknesses to report. We evaluated the impact of giving a simple recommendation of iron supplementation by self-reported use of supplements in GW 28 through a pre-post-test design, not a RCT. We do not know to which extent the women adhered to our recommendations. However, substantially more women reported use of iron supplements in GW 28 among women undergoing this ‘simple intervention’, compared to those not exposed to recommendation, suggesting an effect. In Norway ferrous gluconate, sulphate and fumarate can be bought in grocery stores or pharmacies without prescription. For the majority of women we lack information of the iron dose taken, the frequency and duration of intake, the type of iron supplements as well as of other variables affecting the iron uptake. We assessed the effect of recommending iron supplementation on the women's postpartum status only, and have not evaluated the effect of iron supplementation on the neonates. Further, the number of participants in some ethnic groups was low, and the presence of heterogeneity within relatively broad ethnic groups is possible. As in most studies, we had some loss to follow-up at the postpartum visit, partly due to logistic reasons, but we prioritized ethnic minority women for blood sampling. Further, the collected food-frequency data were used to identify dietary patterns, and we were therefore not able to calculate iron intake. Postpartum haemorrhage was not exactly measured, but based on clinical judgement. On average, the plasma volume increases from about 2.4 L to about 2.8 L from the time before pregnancy to week 19 [39], and we cannot rule out that plasma volume can partly explain some of the differences found during pregnancy. There are no simple biomarkers that provide good estimates of changes in plasma volume [40, 41], however we adjusted for gestational week in our regression models, as a proxy for plasma volume. Furthermore, we have no reason to believe that our explanatory variables of interest will systematically influence the blood volume, and hence affect our results, other than by reducing the precision of our estimates.

### Implications

First, our results lend support to recommend iron supplementation to women during pregnancy to prevent gestational and postpartum ID and anaemia, and indicate that there is a need for an enhanced focus on iron supplementation during pregnancy to be incorporated in high risk groups in antenatal care. Further, our findings also suggest that postpartum ID or IDA needs to be addressed, and postpartum counselling that includes measurement of Hb and SF concentrations are required in high-risk women. In addition, as prophylactic iron supplementation during pregnancy is still a subject of international disagreement, further research to examine the effect on ID and IDA in pregnant women should be undertaken.

## Conclusion

We evaluated the impact of recommending supplements to pregnant women with depleted iron stores, including women in hard-to-reach-groups, and observed an increase of use of supplements and improved iron status from at enrolment to the postpartum visit. Women not exposed to our recommendations, showed a deteriorated iron status postpartum. Further, factors associated with change in iron status were dietary iron intake, use of iron supplements, ethnicity, parity and postpartum haemorrhage.

## Supplementary Information

**Additional file 1.** Flow chart of study participants in the STORK-G multi-ethnic cohort from Oslo 2008-2010.

**Additional file 2. Table S1.** Average SF, sTfR, TBI and Hb concentration in early pregnancy and postpartum, and prevalence of postpartum iron deficiency/anaemia. 

## Data Availability

The editors can access data in de-identified form used in the manuscript, code book, and analytical code upon request. The project manager Anja Brænd, and co-author Line Sletner, will contribute to the access being provided under appropriate conditions. However, research data for this publication include identifying health information subject to confidentiality. It is therefore not possible to share raw data.

## Abbreviations

BMI: Body mass index
CRP: C-reactive protein
CV: Coefficient of variation
CI: Confidence interval
*ECLIA*: Electro-chemiluminescence immunoassay
HbA1c: Glycated haemoglobin
Hb: Haemoglobin
ID: Iron deficiency
IDA: Iron deficiency anaemia
PCA: Principal component analysis
OR: Odds ratio
SF: Serum ferritin
SEP: Socioeconomic position
SD: Standard deviation
sTfR: Soluble transferrin receptor
TBI: Total body iron
WHO: World Health Organization
