# Supplementary figures and images for: The impact of recommending iron supplements to women with depleted iron stores in early pregnancy on use of supplements, and factors associated with changes in iron status from early pregnancy to postpartum in a multi-ethnic population-based cohort
Source: BMC Pregnancy Childbirth. 2023 May 13;23:350. doi: 10.1186/s12884-023-05668-5 (PMC10182636; doi:10.1186/s12884-023-05668-5)

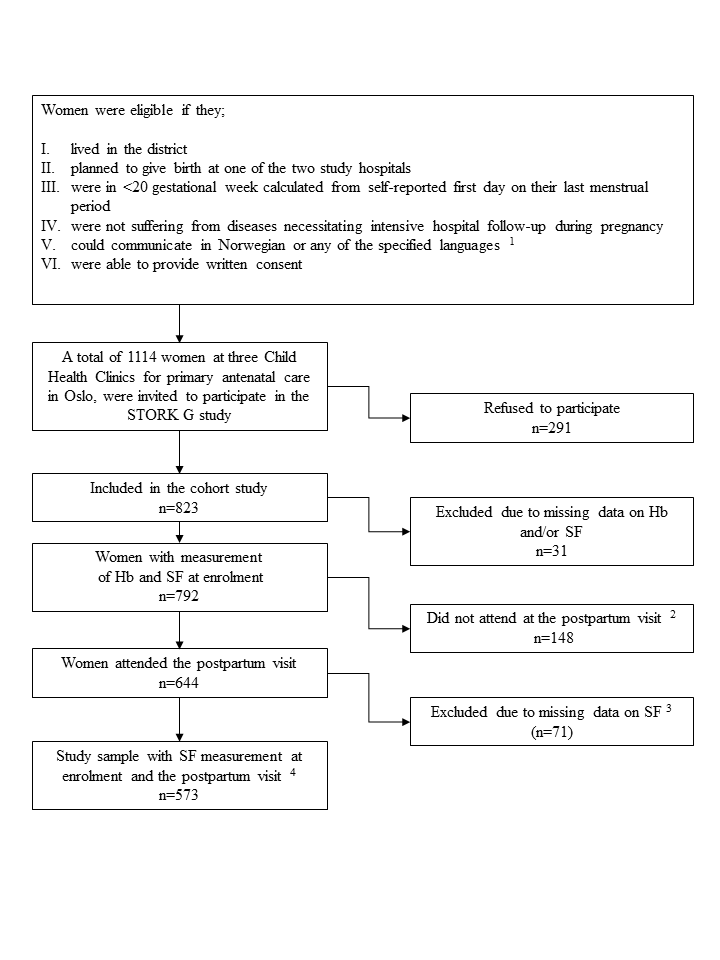

Supplement: Supplementary file 1 — Additional file 1. Flow chart of study participants in the STORK-G multi-ethnic cohort from Oslo 2008-2010. [file 12884_2023_5668_MOESM1_ESM.tif]
