# Supplementary material for: The impact of recommending iron supplements to women with depleted iron stores in early pregnancy on use of supplements, and factors associated with changes in iron status from early pregnancy to postpartum in a multi-ethnic population-based cohort
Source: BMC Pregnancy Childbirth. 2023 May 13;23:350. doi: 10.1186/s12884-023-05668-5 (PMC10182636; doi:10.1186/s12884-023-05668-5)
Supplement: Supplementary file 2 — Additional file 2. Table S1. Average SF, sTfR, TBI and Hb concentration in early pregnancy and postpartum, and prevalence of postpartum iron deficiency/anaemia. [file 12884_2023_5668_MOESM2_ESM.docx]

Additional file 2. Table S1

Average SF, sTfR, TBI and Hb concentration in early pregnancy and postpartum, and prevalence of postpartum iron deficiency/anaemia ^1^

|  | | Total | |  | | SF < 20 μg/L in early pregnancy  (Recommended supplements) | | | | SF ≥ 20 μg/L in early pregnancy  (Not exposed to recommendations) | | | | | | | |
| --- | --- | --- | --- | --- | --- | --- | --- | --- | --- | --- | --- | --- | --- | --- | --- | --- | --- |
|  | |  | |  | | SF < 20 μg/l | | | | SF 20-29 µg/L | | | SF 30-49 µg/L | | | SF 50-69 µg/L | |
|  | | n = 573 | |  | | n = 252 (44) | | | | n = 106 | | | n = 106 | | | n = 46 | |
| *Early pregnancy (mean GW 15*) | |  | |  | |  |  | | |  | | |  | | |  | |
| Median SF (IQR), *μg/L* | | 23 (12, 41) | |  | | 11 (7,15) | | | | 25 (22, 27) | | | 37 (33, 43) | | | 59 (52, 63) | |
| Mean sTfR (95% CI), *mg/L* | | 2.5 (2.4, 2.6) | |  | | 3.1 (2.9, 3.3) | | | | 2.3 (2.1, 2.4) | | | 2.1 (2.0, 2.2) | | | 1.9 (1.7, 2.1) | |
| Mean TBI (95% CI), *mg/kg* | | 4.9 (4.6, 5.3) | |  | | 1.4 (1.1, 1.8) | | | | 5.4 (5.2, 5.6) | | | 7.2 (7.0, 7.4) | | | 9.1 (8.8, 9.4) | |
| Mean Hb (95% CI), *g/dL* | | 12.0 (11.9, 12.1) | |  | | 11.8 (11.6, 11.9) | | | | 12.1 (12.0, 12,3) | | | 12.3 (12.1, 12.5) | | | 12.2 (11.9, 12.5) | |
|  | |  | |  | |  |  | | |  | | |  | | |  | |
| *Postpartum (mean postpartum week 14*) | |  | |  | |  |  |  | | |  | | |  |  |  |  |
| Median SF (IQR), *μg/L* | | 18 (10, 32) | |  | | 16 (9, 31) | | | | 17 (9, 26) | | | 20 (12, 36) | | | 19 (11, 32) | |
| Mean sTfR (95% CI), *mg/L* | | 3.7 (3.5, 3.8) | |  | | 3.9 (3.6, 4.1) | | | | 3.7 (3.4, 4.0) | | | 3.5 (3.2, 3.7) | | | 3.3 (2.9, 3.8) | |
| Mean TBI (95% CI), *mg/kg* | | 2.7 (2.4, 3.0) | |  | | 2.3 (1.8, 2.8) | | | | 2.1 (1.5, 2.8) | | | 3.2 (2.6, 3.9) | | | 3.1 (2.2, 4.0) | |
| Mean Hb (95% CI), *g/dL* | | 12.6 (12.5, 12.6) | |  | | 12.4 (12.3, 12.6) | | | | 12.6 (12.4, 12.8) | | | 12.7 (12.5, 12.9) | | | 12.6 (12.4, 12.9) | |
|  | |  | |  | |  | | | |  | | |  | | |  | |
| Prevalence of ID by SF (<15) ^2^ n (%) | | 224 (39) | |  | | 110 (44) | | | | 43 (41) | | | 36 (34) | | | 19 (41) | |
| Prevalence of ID by SF (<12) ^2^ n (%) | | 167 (29) | |  | | 82 (33) | | | | 37 (35) | | | 25 (24) | | | 13 (28) | |
| Prevalence of ID by sTfR ^3^ n (%) | | 106 (19) | |  | | 61 (25) | | | | 18 (17) | | | 13 (12) | | | 6 (13) | |
| Prevalence of ID by TBI ^4^ n (%) | | 127 (22) | |  | | 65 (28) | | | | 27 (26) | | | 17 (16) | | | 8 (17) | |
| Prevalence of anaemia ^5^ n (%) | | 114 (25) | |  | | 77 (31) | | | | 23 (22) | | | 19 (18) | | | 12 (26) | |

^1^ The STORK-Groruddalen multi-ethnic pregnancy cohort from Oslo, Norway, 2008 – 2010.

*95% CI* 95% confidence interval; *Hb* haemoglobin; *ID* iron deficiency; *SF* serum ferritin; *sTfR* soluble transferrin receptor; *TBI* total body iron.

^2^ Iron deficiency by SF defined as SF concentration <15 μg/L and <12 μg/L.

^3^ Iron deficiency by sTfR defined as sTfR concentration >4.4 mg/L.

^4^ Iron deficiency by TBI defined as TBI concentration <0 mg/kg.

^5^ Anaemia defined as haemoglobin <12.0 g/dL.
